# China Intracranial Aneurysm Project (CIAP): protocol for a registry study on a multidimensional prediction model for rupture risk of unruptured intracranial aneurysms

**DOI:** 10.1186/s12967-018-1641-1

**Published:** 2018-09-26

**Authors:** Junfan Chen, Jian Liu, Yisen Zhang, Zhongbin Tian, Kun Wang, Ying Zhang, Shiqing Mu, Ming Lv, Peng Jiang, ChuanZhi Duan, Hongqi Zhang, Yan Qu, Min He, Xinjian Yang

**Affiliations:** 1 Department of Interventional Neuroradiology, Beijing Neurosurgical Institute, Beijing Tiantan Hospital, Capital Medical University, Beijing, 100050 China; 2 Department of Neurosurgery, Zhujiang Hospital, Southern Medical University, Guangzhou, Guangdong China; 3 Department of Neurosurgery, Xuanwu Hospital, Capital Medical University, Beijing, China; 4 Department of Neurosurgery, Tangdu Hospital, Fourth Military Medical University, Xi’an, Shanxi China; 5 Department of Neurosurgery, West China Hospital, Sichuan University, Chengdu, Sichuan China

**Keywords:** Un-ruptured aneurysms, Rupture risk, Prospective study, Hemodynamics

## Abstract

**Background:**

Ruptured aneurysms, the commonest cause of nontraumatic subarachnoid hemorrhage, can be catastrophic; the mortality and morbidity of affected patients being very high. Some risk factors, such as smoking, hypertension and female sex have been identified, whereas others, such as hemodynamics, imaging, and genomics, remain unclear. Currently, no accurate model that includes all factors for predicting such rupture is available. We plan to use data from a large cohort of Chinese individuals to set up a multidimensional model for predicting risk of rupture of unruptured intracranial aneurysms (UIAs).

**Methods:**

The China Intracranial Aneurysm Project-2 (CIAP-2) will comprise screening of a cohort of 500 patients with UIA (From CIAP-1) and focus on hemodynamic factors, high resolution magnetic resonance imaging (HRMRI) findings, genetic factors, and biomarkers. Possible risk factors for rupture of UIA, including genetic factors, biomarkers, HRMRI, and hemodynamic factors, will be analyzed. The first project of the China Intracranial Aneurysm Project (CIAP-1; chaired by the Department of Neurosurgery, Tangdu Hospital, Fourth Military Medical University, Xi’an, Shaanxi, China) will prospectively collect a cohort of 5000 patients with UIA from 20 centers in China, and collect baseline information for each patient. Multidimensional data will be acquired in follow-up assessments. Statistically significant clinical features in the UIA cohort will also be analyzed and integrated into the model for predicting risk of UIA rupture. After the model has been set up, the resultant evidence-based prediction will provide a preliminary theoretical basis for treating aneurysms at high risk of rupture.

**Discussion:**

This study will explore the risk of rupture of aneurysms and develop a scientific multidimensional model for predicting rupture of unruptured intracranial aneurysms.

*Clinical Trials registration* A Study on a Multidimensional Prediction Model for Rupture Risk of Unruptured Intracranial Aneurysms (CIAP-2), NCT03133624. Registered: 16 April 2017. https://clinicaltrials.gov/ct2/show/NCT03133624

## Background

Intracranial saccular aneurysms are increasingly often being detected in clinical practice in parallel with the increasing frequency of performing computed tomography (CT) and magnetic resonance imaging (MRI). A recent cross-sectional study of community data showed that the prevalence of unruptured intracranial aneurysm (UIA) is as high as 7% [1] among 35–75-year-old individuals in China. The rate of rupture of such aneurysms was almost 1% in a Japanese study [2]. Although the incidence is low, the consequences of aneurysm rupture are serious, the mortality rate in the early stages of hemorrhage reportedly being 40% and the rebleeding rate as high as 60%–70% [2]. Some models for predicting enlargement and rupture of such aneurysms are currently available. However, one of these models is for assessing risk of rupture of bifurcation aneurysms and incorporates only a few indicators [3]. Another large study had some bias that made the external efficiency relatively low [4]. These results and models are difficult to incorporate into clinical practice. It is therefore essential to identify all risk factors and construct a more accurate model for predicting rupture of aneurysms in Chinese people that is simpler to use and more effective in clinical practice.

### Hemodynamic and HRMRI have been gaining increasing attention for predicting rupture of aneurysms

Hemodynamic studies of risk of rupture of aneurysms have shown that turbulence in the aneurysm cavity, strong jet blood flow, and high shear stress can result in changes in the shape of the aneurysm, resulting in a very low velocity region (located at the base of the aneurysm and daughter sac). In areas of low flow velocity and low shear stress, the aneurysm wall cells shrink and degenerate, resulting initially oozing and subsequently in rupture and frank bleeding [5–9]. However, hemodynamic studies have some limitations. For example, some studies have focused too much on computational analysis and lack prospective validation by biological and clinical outcomes. Therefore, studies of hemodynamic characteristics of intracranial aneurysms need to be combined with clinical cohort and pathophysiological studies to provide more comprehensive data.

High resolution magnetic resonance imaging (HRMRI) was first used to diagnose Takayasu arteritis. This modality, which provides resolution accuracy of up to about 78% [10] for the aneurysm wall and surrounding tissue, enabling assessment of the pathology of development of the aneurysm wall, has shown that thickening of the aneurysm wall often denotes infiltration by inflammatory cells, conferring a high risk of rupture [11]. Edjlali et al. [12] found that circumferential aneurysmal wall enhancement (CAWE) is more frequently observed in unstable intracranial aneurysms and can be used as a surrogate for inflammatory activity in the aneurysmal wall. Hu et al. [13] found that wall enhancement consistently and strongly correlates with symptomatic aneurysms. Therefore, wall enhancement that is detected by HRMRI may predict instability of an intracranial saccular aneurysm.

### In the future, progression of aneurysms may be explained on the basis of biochemistry via biomarkers and genetic factors

Indirect evidence from clinical findings has indicated that some genetic factors may be associated with rupture of intracranial aneurysms. Familial aneurysms account for 7%–20% [14] of all aneurysms. Previous studies of familial and sporadic aneurysms have found associations between many regions of genes or chromosomes and presence of aneurysms [15]. However, the results of a large twin study suggest that subarachnoid hemorrhages (SAH) are mainly of non-genetic origin and that environmental variance is greater than genetic variance in individuals with SAH [16]. It is yet to be proved that biological markers and genetic abnormalities can predict SAH in Chinese individuals. Thus, this study will seek to validate target genes and biomarkers that have been identified as possible risk factors by other researchers.

### Clinical factors are important in predicting rupture of aneurysms

There has been ongoing study of the occurrence and rupture of unruptured aneurysms. Studies have shown that up to 85%–90% of ruptured intracranial aneurysms are ≤ 10 mm in diameter [17–20]. It is inappropriate to recommend treatment for all small (< 7 mm) UIAs because treatment-related morbidity and mortality is close to being higher than the morbidity and mortality of aneurysm rupture. The only published lifelong follow-up study found that smoking and female sex are more serious prognostic factors than aneurysm size [21]. Many factors contribute to SAH, the three predominant risk factors being smoking, sex, and blood pressure [22]. Another prospective study [23] found that old age is also a risk factor for aneurysm rupture. The International Study on unruptured intracranial aneurysms [24] found that the 5-year cumulative rupture rates for patients with no history of SAH with aneurysms located in the internal carotid, anterior communicating, anterior cerebral, or middle cerebral arteries were 0%, 2.6%, 14.5%, and 40% for aneurysms smaller than 7 mm, 7–12 mm, 13–24 mm, and 25 mm or greater, respectively, compared with rates of 2.5%, 14.5%, 18.4%, and 50%, respectively, for the same size categories involving the posterior circulation and posterior communicating artery aneurysms. The location and size of an aneurysm thus have an obvious effect on risk of rupture, as has also been shown in another cohort study [2]. The level of evidence in the above two studies was relatively strong. However, because there was a bias in selection, the external validity of the study needs to be verified; additionally, the applicability of the findings of large studies to the Chinese population is unknown. Some studies in China have found that various morphological features of aneurysms are associated with their rupture. These include irregular morphology of a daughter sac and leaf segments, high aspect ratio (ratio of aneurysm height to neck width), and high size ratio (ratio of aneurysm size to parent artery size). Although these studies have enriched our understanding of factors influencing the risk of rupture of aneurysms, some of them are small single center and/or retrospective studies and thus provide a low level of evidence-based data. More importantly, at present, China does not have a high-level prediction model that integrates these factors. Thus, we plan to carry out a large prospective multicenter cohort study with wide coverage to investigate rupture-related factors in China.

## Methods and design

The Study on a Multidimensional Model for Predicting Risk of Rupture of Unruptured Intracranial Aneurysms (CIAP-2) is the second project of the China Intracranial Aneurysm Program and is managed by Beijing Neurosurgical Institute and Beijing Tiantan Hospital), which manages a prospective observational registry of patients with UIA who were enrolled in our 20 centers in China during the first CIAP. It is sponsored by the National Key Research Development Program of China. This study has been designed as a prospective, observational registry and the treatment of patients will not be influenced by their participation in this observational study. This study adheres to the ethical principles of the Declaration of Helsinki and Human Biomedical Research Ethical Issues and Policy Guidance. The Institutional Review Board of Beijing Tiantan Hospital, Capital Medical University has approved the study (KY2017-017-01). We will inform participating patients or their relatives that the privacy of their data will be protected. These know they can withdraw from the study at any time. The study has been registered at https://clinicaltrials.gov/ct2/show/NCT03133624.

### Aims

**Fig. 1:**
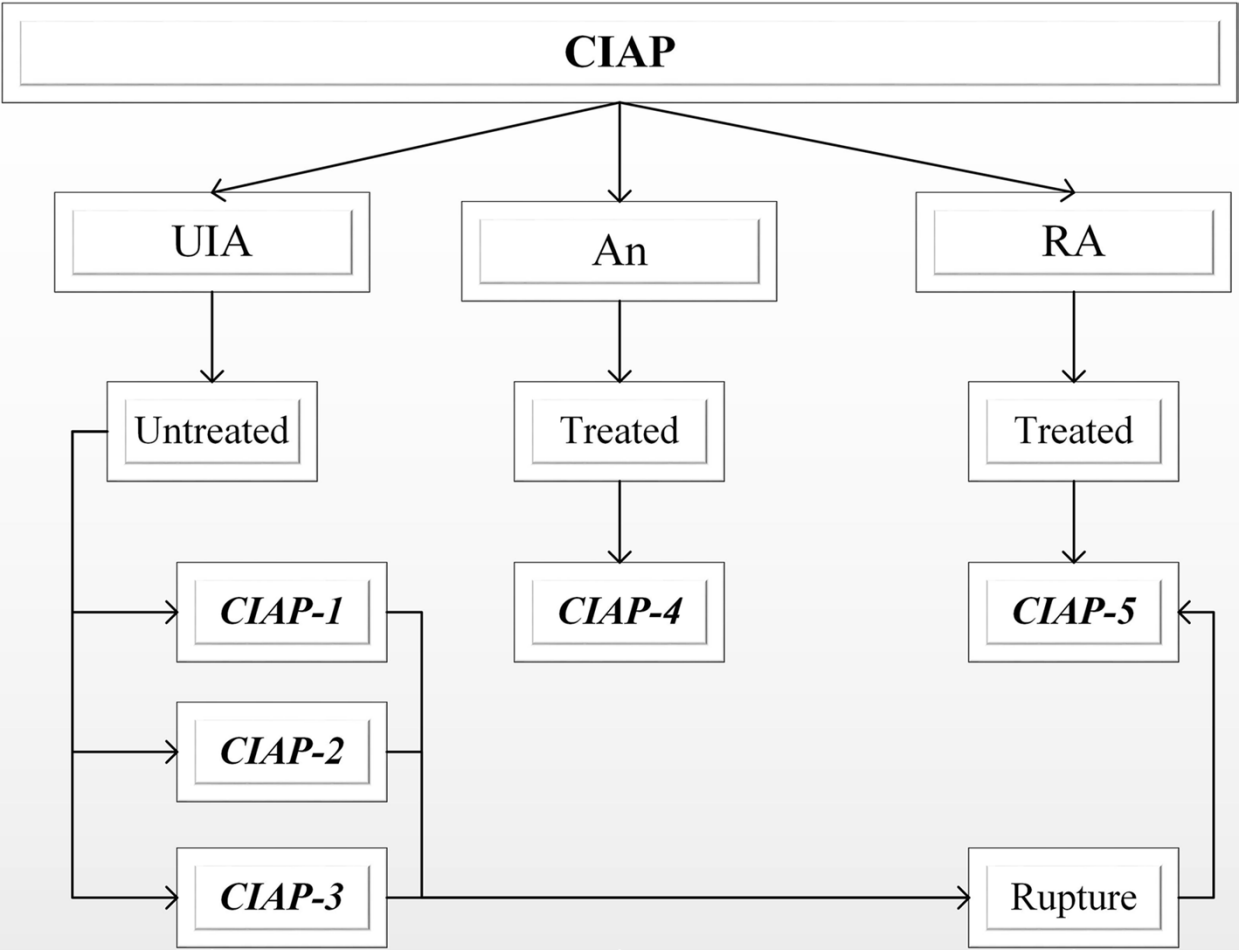
Flow chart of the CIAP study. *An* aneurysm, *RA* ruptured aneurysm, *UIA* unruptured intracranial aneurysm

CIAP is a multicenter, large prospective study of intracranial aneurysms that has five different facets: CIAP-1 is for setting up and managing a UIA queue of 5000 individuals and calculating the annual rate of rupture of those aneurysms; CIAP-2 for exploring risk factors associated with aneurysm rupture and developing a multidimensional model for predicting aneurysm rupture; CIAP-3 for exploring antithrombotic therapy in patients with UIA complicated by ischemic cardio-cerebrovascular diseases; CIAP-4 for comparing treatment options for UIA-intervention and craniotomy, the study protocol of CIAP-4 has been published [25]; and CIAP-5 is for development of standardized treatments for early-stage bleeding from aneurysms. CIAP-1 is responsible for the management of the UIA queue. This is for the second observational study. An overview of the CIAP design is presented in Fig. 1.

### Design

**Fig. 2:**
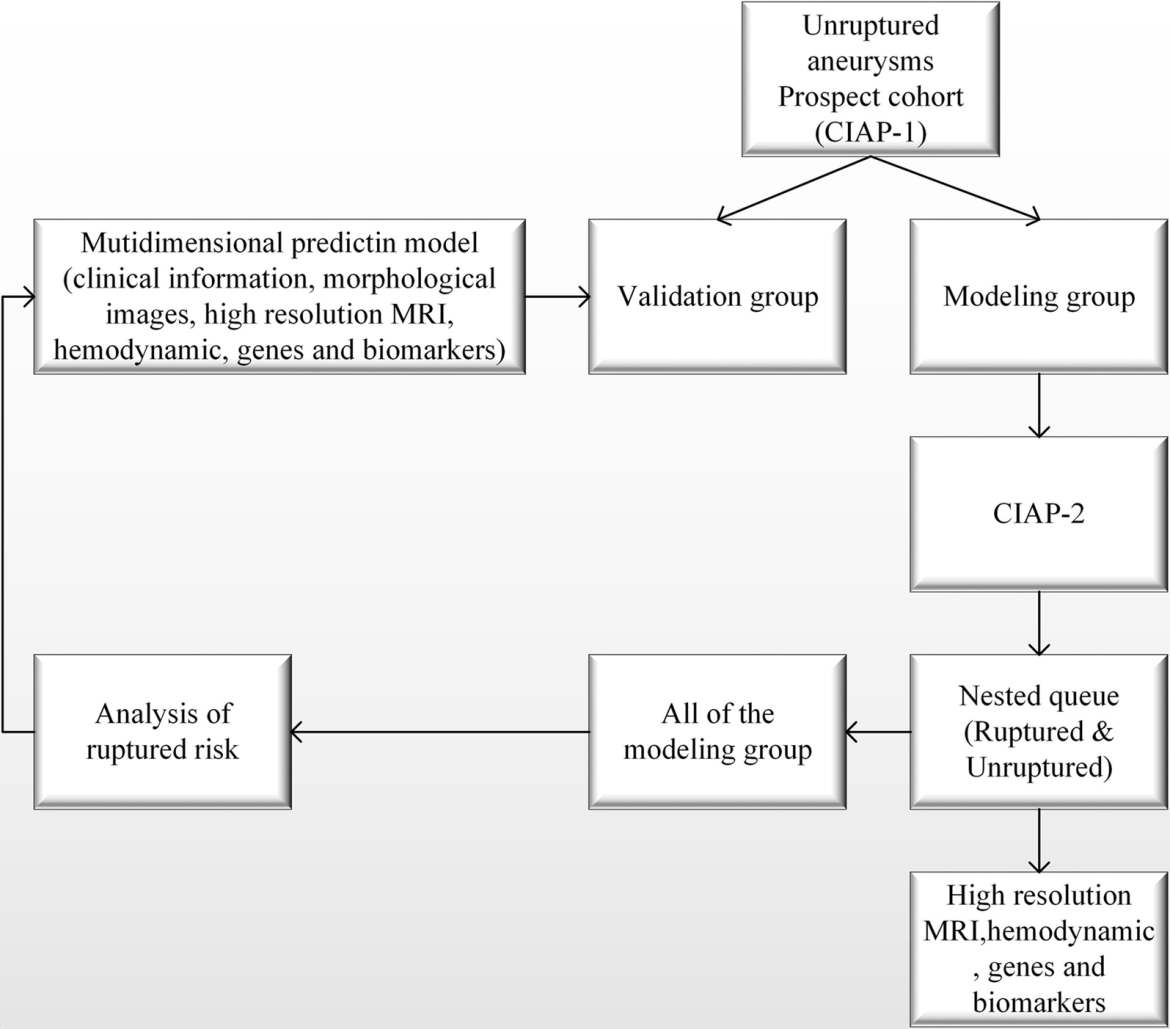
Flow chart showing development of multidimensional model for prediction bleeding from previously unruptured aneurysms. *MRI* magnetic resonance imaging

Individuals in the UIA follow-up cohort will be allocated to two groups, a modeling and a validation group, in a ratio of 2:1. Taking the annual rupture rate as 1% [2], the rate of loss would be less than 20%. The data will be analyzed statistically in the 5th years, the expectation being that at least 130 patients’ aneurysms will rupture in the first 4 years. The modeling group will be used to establish the risk of UIA rupture and compare relevant clinical information, radiological images, hemodynamic variables, genetic factors, HRMRI findings, and biomarkers [12, 13, 26–37] between the rupture and non-rupture groups to identify the risk factors for rupture. These will then be incorporated into a multi-dimensional model for predicting risk of UIA rupture. The validation group will then be used to validate that model (Fig. 2).

### Recruitment of participants

Inclusion criteria

- At least one UIA that has not been treated and has been confirmed by imaging: computed tomography angiography (CTA), magnetic resonance angiography (MRA), and/or digital subtraction angiography (DSA).
- The ability to live independently and a modified ranking score ≤ 3.
- Older than 14 years.
- Patient or relatives have given written informed consent.

Exclusion criteria

- Subarachnoid hemorrhage of unknown origin.
- Presence of other intracranial vascular malformations, such as a cerebral arteriovenous malformation (AVM) or cerebral arteriovenous fistula (AVF).
- Intracranial and other malignant aneurysms.
- Traumatic, bacterial, or dissecting aneurysm.
- Presence of severe mental illness preventing communication.
- Presence of other diseases or poor general condition with expected survival of less than 1 year.
- Participant in another similar research program.
- Refusal to attend for follow-up.

### Data collection and management

The following patient characteristics will be collected and recorded: (1) basic clinical characteristics; (2) relevant data concerning personal and family history; (3) relevant imaging findings; (4) findings of head HRMRI, computational fluid dynamics (CFD) testing, and relevant genetic testing; (5) relevant laboratory findings; and (6) neurological function-related scores on admission or first visit. The following characteristics of aneurysms will be collected and recorded: size, neck size, dome-to-neck ratio, dome-to-artery ratio, shape, location, clinical presentation, and number of aneurysms.

The follow-up data collected will comprise: (1) frequency and number of follow-up assessments; (2) findings on general evaluation; (3) deaths; (4) imaging findings; (5) UIA growth (assessed by repeated imaging to avoid treatment bias); and (6) life-style changes. Throughout, neurobehavioral-related scores will include: (1) Glasgow Coma Scale scores; (2) improved RANKIN scale scores (somatic changes); and (3) Mini-Mental State Examination scores (psychological changes).

The participants will initially be followed up three times: at 90 ± 14 days, 180 ± 30 days, and 365 ± 30 days and at least annually for a minimum of 5 years thereafter. Participation in the study will end if rupture occurs. Patients with SAH will be transferred to the CIAP-5 project (a study of emergency treatment of aneurysmal subarachnoid hemorrhage; CIAP-5, the fifth project of the China Intracranial Aneurysm Project, is managed by Xuanwu Hospital, Capital Medical University, Beijing, China). In addition, patient’s relatives are requested to report sudden deaths to the study group. In such instances, autopsies will immediately be organized in study hospitals and tissue samples of ruptured aneurysms will be supplied to the project for analysis, subject to the informed consent of the participants’ relatives.

Formal training, including investigator training and familiarization with the study protocol, will be initiated. An inspection team of experts drawn from various organizations will follow participants up with the specified regular monitoring until the end of the study period. All data will be collected on paper Case Report Forms (CRF), then input into Electronic Data Capture (EDC) for management, and retained for 5 years after the end of the study. The accuracy and rigorousness of the data will be checked in a timely manner.

This study will incorporate nested screening of 500 individuals from cohort 1 (CIAP-1). Recruitment for this study started in September 2016. The estimated primary completion date will be in December 2020. The project will produce preliminary results in the 5th year. After the end of the 5th year, we will have a longer follow-up visit to the patients who have been in the group.

### Model building (first set of UIA queues)

1. The following possible or established risk factors for UIA will be collected and analyzed: age; sex; blood pressure, smoking, alcohol consumption, hyperlipidemia, diabetes mellitus, and family history of subarachnoid hemorrhage, intracranial aneurysm, and autosomal dominant diseases (polycystic kidney, Marfan syndrome, collagen dysplasia, etc.)
2. The following imaging data will be collected and analyzed: shape, location, size, aspect ratio and odds ratio of aneurysms, morphological factors such as the angle of the aneurysm jet, and known risk factors for rupture.
3. In our experience, follow-up angiography alone does not adequately predict the outcome whereas HRMRI is a more useful means of following these lesions [38]. We have also found and reported that aneurysm wall enhancement on HRMRI predicts instability of an intracranial aneurysm [39]. HRMRI will be performed on patients enrolled in the UIA follow-up cohort at the 20 participating centers and HRMRI-identified aneurysm wall enhancement assessed as a possible predictor of UIA rupture. Two-dimensional short-axis images of the aneurysms generated from the registered pre-and post-contrast T1w-SPACE images will be used to identify wall enhancement [13].
4. To identify risk factors, the nested cohort study will incorporate the following: incidence of aneurysm rupture in the follow-up cohort, the definition of UIA recently rupture before the duration of follow-up was high risk of rupture of UIA, acquisition of imaging data for hemodynamic calculations; according to the aneurysm ratio of 1:2 UIA (rupture vs non-rupture), and analysis of differences in hemodynamic variables, genetic factors, and biomarkers.
  A. Analysis of hemodynamic variables: A three-dimensional shape will be created and image data from computed tomographic angiography, magnetic resonance angiography, or DSA saved as DICOM files. Once the 3D model has been reconstructed, it will be saved in an STL file format. A rigid wall in the parent vessel and aneurysm will be assumed in the simulation of hemodynamics. We will start by using ANSYS ICEM CFD (Materialise, Leuven, Belgium) to build a finite element model, then perform a simulation on ANSYS CFX software (Materialise), and finally use ANSYS CFX POST postprocessing to calculate Wall Shear Stress (WSS), maximum wall shear stress (WSSmax), low WSS areas, steady state of blood flow, and so on. Our team has already identified that these factors are associated with the stability of aneurysms [40–42].
  B. Gene and biomarker analysis: Blood samples will be collected from all participants and specimens of the aneurysm wall from patients who undergo craniotomy. Target chromosomes will be tested for blindly. Target genes and biomarkers identified as possibly relevant by the latest international research will be tested. Differences in genes and biomarkers between rupture and non-rupture groups will be compared to identify rupture-related factors.
5. Risk factors for rupture, including hemodynamic variables, genes, and biomarkers will be calculated in a nested cohort study of the entire first cohort (CIAP-1: the first project of the China Intracranial Aneurysm Project and is managed by Tangdu Hospital, Fourth Military Medical University). A multi-dimensional model for predicting UIA rupture and bleeding that incorporates clinical, imaging, HRMRI data, and so on will be constructed by using a statistical model.
6. Model validation: After establishment of the model, the second group of the follow-up cohort will be used for blind verification.
7. Comparative analyses of histopathology of aneurysm walls: When participants undergo surgery for ruptured intracranial aneurysms, the sites of rupture will be identified intraoperatively and specimens of the aneurysm wall will be removed and preserved in formalin. The location of rupture, pathological damage, hemodynamic variables, HRMRI findings, and relevant biomarkers will be analyzed to identify differences between unruptured and unruptured aneurysms. Relationships between pathological damage, hemodynamic variables, and HRMRI features of the aneurysm wall will also be assessed.

## Statistical methods

### Total sample size

CIAP-1: Calculation of the required sample size using an expected annual rupture rate of 1%, allowable error of d = 0.6%, and alpha = 0.05 on two-sided testing indicated that 1173 patients need to be enrolled per year, that is, 4692 over 4 years. An annual rupture rate of 1% [2] results in a loss rate of less than 20%. A reported hemodynamic index (complex flow patterns OR = 4.7) and HRMRI index (aneurysmal wall enhancement OR = 3.05 for ruptured versus unruptured aneurysm) also indicate that this sample size would be sufficient [12, 43]. Given that genetic factors have not yet been evaluated [35], our genetic testing will assess previously reported possible genetic indicators of prognosis; additionally, target genes and biomarkers will be selected according to the latest research progress in the future. In the 5th year of the study, we will analyze the data. As previously mentioned, at least 130 aneurysms are expected to rupture in the modeling group in the first 4 years. Allowing for a loss rate of 20%, the study will need to enroll 500 individuals.

### Expected loss rate (less than 20%)

The expected loss includes all patient data excluded from the main analysis, usually because of a serious breach of the study protocol by the principal investigator (affecting the evaluation of the primary endpoint of effectiveness). Possible such breaches include failure to meet the inclusion/exclusion criteria; failure to follow the prescribed schedule; follow-up visits not occurring within the specified time limits, and concomitant treatments that may affect incidence of rupture. However, excellent clinical follow-up may achieve a drop-out rate of as little as 5%.

### Statistical analysis methods

#### Statistical analysis of clinical data

Continuous variables will be expressed as mean and standard deviation analyzed by one-way anova. Categorical variables will be expressed as quantity (rate) and compared using χ^2^ tests. Continuous variables that are not normally distributed will be expressed as medians and quantile intervals and compared using the Mann–Whitney nonparametric test. P < 0.05 will denote a significant difference.

#### Evaluation of risk factors for aneurysms

Multiple regression analysis will be used to calculate OR values for high risk factors, including life habits and clinical and epidemiological data. Confidence intervals of 95% and cut-off values for sensitivity and specificity for predicting occurrence of an aneurysm rupture will be determined by receiver operating curves (ROCs). Next, the model will be used to assess risk of aneurysm rupture in the validation group and thus determine the model’s efficacy and accuracy. Finally, the model will be adjusted as indicated and established.

## Discussion

The study will comprise only Chinese individuals and incorporate Korja et al.’s recommendations for research on UIAs [44]. This study will be the first multicenter prospective registry study of a multidimensional model for predicting aneurysm rupture in China. In addition to the previous study of baseline data, this study will also explore hemodynamic factors, imaging factors, genomics, and biomarkers in more detail with the aim of making it more applicable to clinical situations. The assumption of rigid wall in the parent vessel and aneurysm may introduce bias in our simulation results. Image data and biochemical indicator of different centers will be standardized; however, data biases associated with different types of imaging machines and different detection methods are inevitable. Additionally, because this research includes only Chinese individuals, the results will be more applicable to Chinese people than to others; the applicability to other populations is unknown.

## Abbreviations

CIAP: China Intracranial Aneurysm Project
CFD: computational fluid dynamics
CRF: Case Report Form
CT: computed tomography
CTA: computed tomography angiography
DSA: digital subtraction angiography
EDC: Electronic Data Capture
HRMRI: high resolution magnetic resonance imaging
MRA: magnetic resonance angiography MRI: magnetic resonance imaging
SAH: subarachnoid hemorrhage
UIA: unruptured intracranial aneurysm
